# Post-surgery Rehabilitative Intervention Based on Imitation Therapy and Mouth-Hand Motor Synergies Provides Better Outcomes in Smile Production in Children and Adults With Long Term Facial Paralysis

**DOI:** 10.3389/fneur.2022.757523

**Published:** 2022-05-11

**Authors:** Elisa De Stefani, Anna Barbot, Cecilia Zannoni, Mauro Belluardo, Chiara Bertolini, Rita Cosoli, Bernardo Bianchi, Andrea Ferri, Francesca Zito, Michela Bergonzani, Arianna Schiano Lomoriello, Paola Sessa, Pier Francesco Ferrari

**Affiliations:** ^1^Unit of Neuroscience, Department of Medicine and Surgery, University of Parma, Parma, Italy; ^2^Child and Adolescent Neuropsychiatry–NPIA District of Scandiano, AUSL of Reggio Emilia, Reggio Emilia, Italy; ^3^Operative Unit of Maxillo-Facial Surgery, Head and Neck Department, University Hospital of Parma, Parma, Italy; ^4^Section for Cognitive Systems, DTU Compute, Technical University of Denmark, Kongens Lyngby, Denmark; ^5^Department of Developmental and Social Psychology, University of Padova, Padova, Italy; ^6^Padova Neuroscience Center (PNC), University of Padova, Padova, Italy; ^7^Institut des Sciences Cognitives Marc Jeannerod, CNRS, Université de Lyon, Lyon, France

**Keywords:** hand-mouth synergies, smile surgery, free gracilis muscle transfer, Moebius syndrome, mirror neurons, action observation (AO)

## Abstract

Rehabilitation after free gracilis muscle transfer (smile surgery, SS) is crucial for a functional recovery of the smiling skill, mitigating social and psychological problems resulting from facial paralysis. We compared two post-SS rehabilitation treatments: the traditional based on teeth clenching exercises and the FIT-SAT (facial imitation and synergistic activity treatment). FIT-SAT, based on observation/imitation therapy and on hand-mouth motor synergies would facilitate neuronal activity in the facial motor cortex avoiding unwanted contractions of the jaw, implementing muscle control. We measured the smile symmetry on 30 patients, half of whom after SS underwent traditional treatment (control group, CG mean_age_ = 20 ± 9) while the other half FIT-SAT (experimental group, EG mean_age_= 21 ± 14). We compared pictures of participants while holding two postures: maximum and gentle smile. The former corresponds to the maximal muscle contraction, whereas the latter is strongly linked to the control of muscle strength during voluntary movements. No differences were observed between the two groups in the maximum smile, whereas in the gentle smile the EG obtained a better symmetry than the CG. These results support the efficacy of FIT-SAT in modulating the smile allowing patients to adapt their smile to the various social contexts, aspect which is crucial during reciprocal interactions.

## Introduction

Facial nerve paralysis is the inability to move the muscles that control smiling, blinking, and other facial movements. When the facial nerve is non-functioning the face muscles do not receive the necessary signals in order to produce the appropriate contraction. This results in paralysis of one or both sides of the face and the loss of facial expression that has serious implications for a patient's physical and psychological wellbeing (1). The treatment of facial paralysis is determined by the ethiology and by what portions of the face are affected. In patients presenting congenital or acquired long term facial paralysis, facial reanimation surgery (smile surgery, SS) is the optimal option in order to restore a dynamic smile (2–6). During SS, also known as functional muscle transfer (2, 7), a new muscle is inserted into the paralyzed face to shape a smile. Free muscle transfer allows the maxillo surgeon to create a dynamic reanimation of the paralyzed face and the creation of a voluntary smile (2, 3, 5, 6). Depending on the type of paralysis (unilateral or bilateral), patients undergo one or two muscle transplants at least 6 months apart. The most common muscle used for SS is the *gracilis* muscle: a portion of this muscle is taken from the patients' leg with its own nerve and blood vessels and transferred into the face. Here it is connected to the masseteric nerve (derived from a branch of the trigeminal nerve) which is responsible for activating the bite *via* the masseter muscle (2, 4, 6). After surgery, the recovery of the aesthetic smile is the main aspect to be taken into consideration to improve the patients' quality of life and substantial effort is made by clinicians to improve rehabilitation protocols for obtaining spontaneous and symmetrical activation of the smile (1, 8). In literature, there is a high degree of heterogeneity in facial rehabilitation approaches described for adults with acquired facial paralysis (9, 10), which were subsequently adapted to the rehabilitation of patients with congenital paralysis (both adults and children). Some studies reported muscle strengthening exercises alone (11) or associated with biofeedback (12, 13) (using mirror to reflect patients' orofacial movements). Additional forms of rehabilitation make also use of massage, stretching or electrical stimulation (10). To date the rehabilitation treatment currently in use involves mirror feedback and teeth clenching to recruit the transplanted muscle/s (14). Patients trigger a bite to create a smile and use the mirror to observe their movement and correct it when necessary. This (traditional) treatment, although very effective in quickly recruiting muscle/s, does have some disadvantages. First of all, patients report that they do not like looking at their face in the mirror and soon interrupt the treatment. Secondly, the use of teeth clenching results in unpleasant lips movements and poor postures that are difficult to correct. In addition to this, patients have little awareness of the muscle/s contraction force (15).

We propose a new neurorehabilitation treatment (FIT-SAT) that aims at recruiting the transplanted muscle without grinding teeth (8, 15). FIT-SAT is based on the principle of action observation (AO) and aims at enhancing motor learning and promote neural reorganization in patients after SS. AO practice commonly includes the observation of an action and its subsequent execution (16–18). A large number of studies suggested some benefits of AO such as facilitating motor function after arms impairments allowing patients to practice movements (17). AO is based on the well-known mirror neuron system (MNS) which leads to recruitment of functionally interconnected cortical structures coupling action execution and observation (19–21). From a theoretical point of view, mirror mechanism alludes to the activation of motor-related areas not only when an action is performed, but also when the action is observed (19, 20, 22, 23). On the basis of this mechanism, FIT-SAT exploits motor and premotor activations present during smile observation to facilitate the activation of the corresponding cortical motor representation. The FIT-SAT treatment also requires that patients simultaneously close their hand during smile execution (hand/mouth synergies) with the aim of facilitating the recruitment of the motor programs of opening the mouth involved in the smile, avoiding teeth contraction (8, 15). The concept of synergy indicates functional modules that simplify the control of complex motor activation patterns by combining elemental movements that are represented at different brain locations (24–26), but adjacent to each other with partially overlapping fields (e.g., the hand and mouth cortical motor representations). Such anatomical organization is functionally meaningful, for example during the unfolding of complex actions involving multiple effectors (such as grasping food with the hand to bring it to the mouth for eating it). Hand/mouth synergies probably are the most well-known and studied example of synergies because their frequency of use and the high ethological value. Notably, the electrical stimulation of specific sites of the precentral gyrus in the right hemisphere of monkey can cause left hand closed in a grip posture together with mouth opening (27). This stimulation-induced sequential movements are very similar to that voluntary performed by the monkey under ethological conditions. Similar results were obtained in patients undergoing awake brain surgery: the stimulation of the premotor region triggered overt mouth and contralateral limb movements (24). Behavioral studies on humans have further confirmed the existence of these synergies by highlighting how the closing of the hand (precision or power grip) influenced the opening of the mouth (20, 28). Thus, exploiting hand-mouth synergies present at the cortical level (24, 27) we expected that closing the hand during the smile would facilitate mouth aperture.

In this study we tested the efficacy of the FIT-SAT in comparison to traditional treatment in controlling the contraction force of the transplanted muscle/s, thus obtaining a modulated and symmetrical smile. For this reason, patients were photographed at the end of the treatment and the symmetry of their smiles was analyzed in three conditions: rest position (baseline), gentle and maximum smile. Unlike the maximum smile, the gentle smile is characterized by a small excursion of the lips which results in a subtle control of the muscle contraction force. Compared to the traditional treatment, we expected a better performance in the gentle smile condition, the one which requires better awareness and fine motor control.

## Materials and Methods

### FIT-SAT Description and Participants

**Figure 1:**
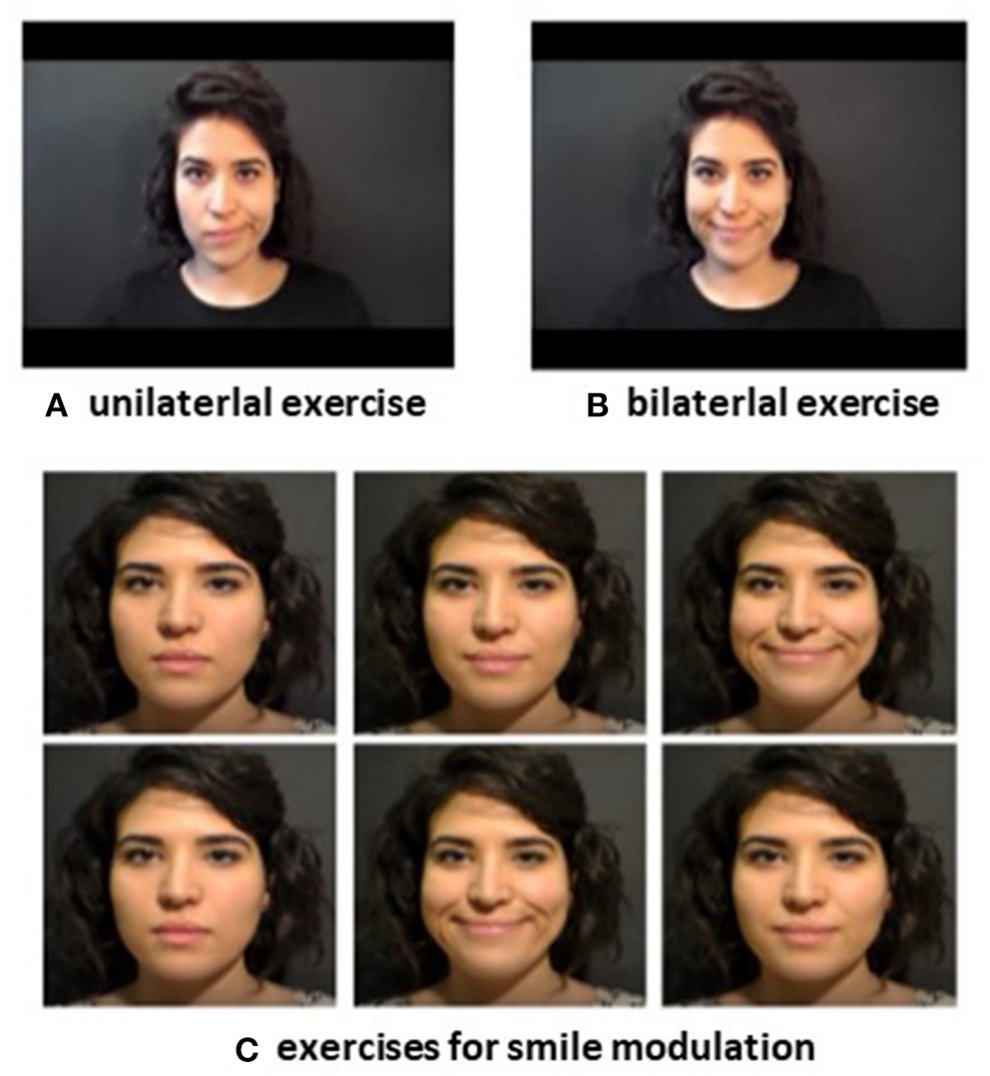
FIT-SAT protocol consists in an action-observation protocol and it includes videos in which an actor performs smiles (facial imitation treatment, FIT) and provides instructions concerning both the co-activation of hand closed as a fist (synergistic activity treatment, SAT) and the number of repetitions that the patients will perform. Specifically, the FIT-SAT protocol consists in two phases: unilateral and bilateral phases. In unilateral phase the goal is to support patients in recruiting the transplanted muscle through unilateral exercises. The first unilateral phase starts about 8 weeks after the surgery, when the patients begin to recruit the transplanted muscle. It consists in the observation of a 3 s smile produced by an actor on a video and the instruction for the subsequent patient's synergetic hand-mouth contraction **(A)**. The task of the patients is to imitate the actor's smile and, while they are smiling, simultaneously clench their fist. Each patient starts the daily session with five repetitions repeated for three times. Progressively, further repetitions are gradually included until the patient is able to perform at least ten successive repetitions and to maintain the posture for at least 3 s. This second phase starts after a clinical evaluation performed by the speech therapist who assesses the patient's ability to recruit the muscle without hand contraction. In the bilateral phase the goal is to achieve a symmetric smile and to be capable to modulate it through bilateral exercises **(B)**. Bilateral exercises include modulation tasks in which the patient is asked to perform maximum and small (gentle) smiles in order to train and control the contraction force of the transplanted muscle/s **(C)**.

The FIT-SAT is performed at home for at least 6 months under the supervision of an expert speech therapist that modulates the exercises to be performed (for a detailed description of the treatment, [see (6, 15)]. Specifically, the FIT-SAT includes the observation and execution of corner-of-the-mouth smile (29, 30) accompanied by the closing of the hand and it is divided into two phases: the first phase begins as soon as the patient starts to recruit the transplanted muscle and aims at increasing muscle strength with unilateral exercises. This phase of the rehabilitation consists in watching a series of video clips of an actor performing unilateral smiles that patients have to observe and reproduce (Figure 1A). The second phase can start only when patients are able to perform at least three consecutive repetitions of the unilateral movement, maintaining the posture for at least three seconds (15). Only at this point of the rehabilitation process the second phase begins. Patients perform bilateral exercises in order to coordinate the two half-sides of the face. Moreover, modulation tasks are included in which to the patient is asked to perform maximum and small (gentle) smiles (Figures 1B,C). These exercises aim at training and controlling the contraction force of the transplanted muscle/s. The second phase of the FIT-SAT ends when the speech therapist determines that the patient is able to synchronize the contraction of both sides in order to obtain a harmonious movement and a natural smile.

Thirty patients (mean age = 20.7 ± 11.4 years) with congenital and acquired facial paralysis were included. All patients underwent SS procedure in the Division of Maxillo-Facial Surgery, Head and Neck Department, of the University Hospital of Parma. Specifically, all patients underwent *gracilis* neuromuscular transplantation re-innervated with masseteric nerve.

The inclusion criteria were: (1) unilateral or bilateral congenital facial paralysis (Moebius syndrome); (2) unilateral established long term facial palsy (>18 months) (31); (3) facial animation *via* gracilis neuromuscular transplantation and re-innervation with ipsilateral masseteric nerve; (4) Post SS rehabilitation with traditional treatment (control group, CG). (5) Post SS rehabilitation with FIT-SAT (experimental group, EG); (6) absence of congenital hands malformations; (7) absence of any psychiatric or physical illness at the time of participation; (8) age >6 years. Patients' characteristics are reported in Table 1.

**Table 1:** Patient classification: demographics and clinical characteristics of patients.

**ID_subject**	**Group**	**Type of paralysis**	**Side of paralysis**	**Age**	**Sex**	FDI_physical function^*^	FDI_social function^*^
ID_01_EG	Experimental group	Congenital	Right	14	M	60	64
ID_02_EG	Experimental group	Acquired	Right	28	F	85	84
ID_03_EG	Experimental group	Congenital	Right	16	M	80	40
ID_04_EG	Experimental group	Congenital	Bilateral	14	F	75	84
ID_05_EG	Experimental group	Congenital	Right	41	F	77	68
ID_06_EG	Experimental group	Acquired	Left	49	F	70	72
ID_07_EG	Experimental group	Congenital	Right	9	F	95	32
ID_08_EG	Experimental group	Congenital	Right	35	F	65	76
ID_09_EG	Experimental group	Congenital	Left	10	F	80	60
ID_10_EG	Experimental group	Congenital	Bilateral	37	F	85	56
ID_11_EG	Experimental group	Congenital	Bilateral	20	M	50	84
ID_12_EG	Experimental group	Congenital	Right	20	F	90	76
ID_13_EG	Experimental group	Congenital	Bilateral	8	F	55	88
ID_14_EG	Experimental group	Congenital	Right	9	F	100	68
ID_15_EG	Experimental group	Congenital	Bilateral	8	M	75	68
ID_16_CG	Control group	Congenital	Right	17	F	60	24
ID_17_CG	Control group	Congenital	Right	14	F	85	92
ID_18_CG	Control group	Congenital	Left	27	F	90	100
ID_19_CG	Control group	Congenital	Bilateral	27	M	85	68
ID_20_CG	Control group	Congenital	Left	14	F	100	100
ID_21_CG	Control group	Congenital	Bilateral	19	M	85	92
ID_22_CG	Control group	Congenital	Bilateral	13	F	90	72
ID_23_CG	Control group	Congenital	Bilateral	22	F	95	72
ID_24_CG	Control group	Congenital	Bilateral	14	M	87	82
ID_25_CG	Control group	Congenital	Bilateral	17	F	90	80
ID_26_CG	Control group	Congenital	Right	18	M	95	92
ID_27_CG	Control group	Congenital	Right	15	F	90	100
ID_28_CG	Control group	Congenital	Left	7	F	40	64
ID_29_CG	Control group	Congenital	Bilateral	36	M	65	76
ID_30_CG	Control group	Acquired	Left	42	M	55	56

* *Scores of physical and social subscales are reported transformed to a score on a 100-point scale. A value 100 indicates unimpaired physical or social/wellbeing function (32)*.

Written consent was obtained after full explanation of the research procedure, in agreement with the Declaration of Helsinki. The treatment was approved by the Joint Ethics Committee of the Parma Department of Medicine and Surgery and of the Parma Hospital on 12nd October 2016 (Prot. 34819). Functional limitations and quality of life have been evaluated using the Italian version of Facial Disability Index (FDI).

### Procedure and Data Acquisition

High definition frontal-view photographs were captured with a digital camera Canon E0S 100D (18–55 mm lens, 18 megapixel) at a distance of 60 cm from the participants' face. We photographed the participants while holding the following postures: rest (baseline), gentle and maximal smile (6). The photos were uploaded and analyzed using Emotrics [Emotrics Software, Mass Eye and Ear, Boston, MA (33)] an open source software based on a machine learning algorithm that calculates a full set of measurements relevant to quantify facial symmetry (33).

The software created 68 facial landmarks that could be checked and corrected by the experimenter. The software estimated a set of facial measurements to assess facial symmetry using the position of these landmarks. Emotrics measurements were: commissure excursion (CE, the distance from the midline of the vertical/lower lip vermilion junction to the oral commissure) (6), commissure height deviation (CHD, vertical distance between the horizontal plane of the left and the right oral commissure), upper/lower lip height deviation (ULHD/LLUD, vertical distance between horizontal planes taken from the upper/lower lip vermillion border points where they intersect with a vertical plane taken midway between the mid-vertical and the oral commissure) and smile angle [SA, the angle between the horizontal plane at the midline vertical/lower lip vermilion junction and the oral commissure (6)].

We have also evaluated physical and social functions using the Italian version of Facial Disability Index (IT-FDI) (32), the first validated questionnaire in Italian for quality of life in patients with facial palsy (32). The IT-FDI is a questionnaire (10-item) divided into TWO parts: physical function and social/wellbeing function subscales. The first one investigates different functional problems such as eating, drinking, speaking, etc. The second subscale investigates quality of life and limitations due to the paralysis. Participants responded to each item on a six-point scale forces choice, ranging from complete disability to absence of disability.

### Statistical Analysis

All data were tested for normality using a Shapiro-Wilk's test and Levene's test to examine normality and homogeneity of variance (*p* > 0.05). Analyses of covariance (ANCOVAs) were performed adjusted for participants' age to test for differences in symmetry between groups (EG vs. CG). We were interested to test for any statistically significant differences on Emotrics measurements (CE, CHD, ULHD/LLUD and SA) and FDI scores between the two treatments. As no significant differences in baseline (rest position) were observed between groups, each kinematic parameter was expressed as a difference between the topic smiles (gentle and maximum) and their baseline. Statistical analyses were performed using Jamovi (version 1.6.9.0). Statistical significance was considered at *p* < 0.05.

## Result

**Figure 2:**
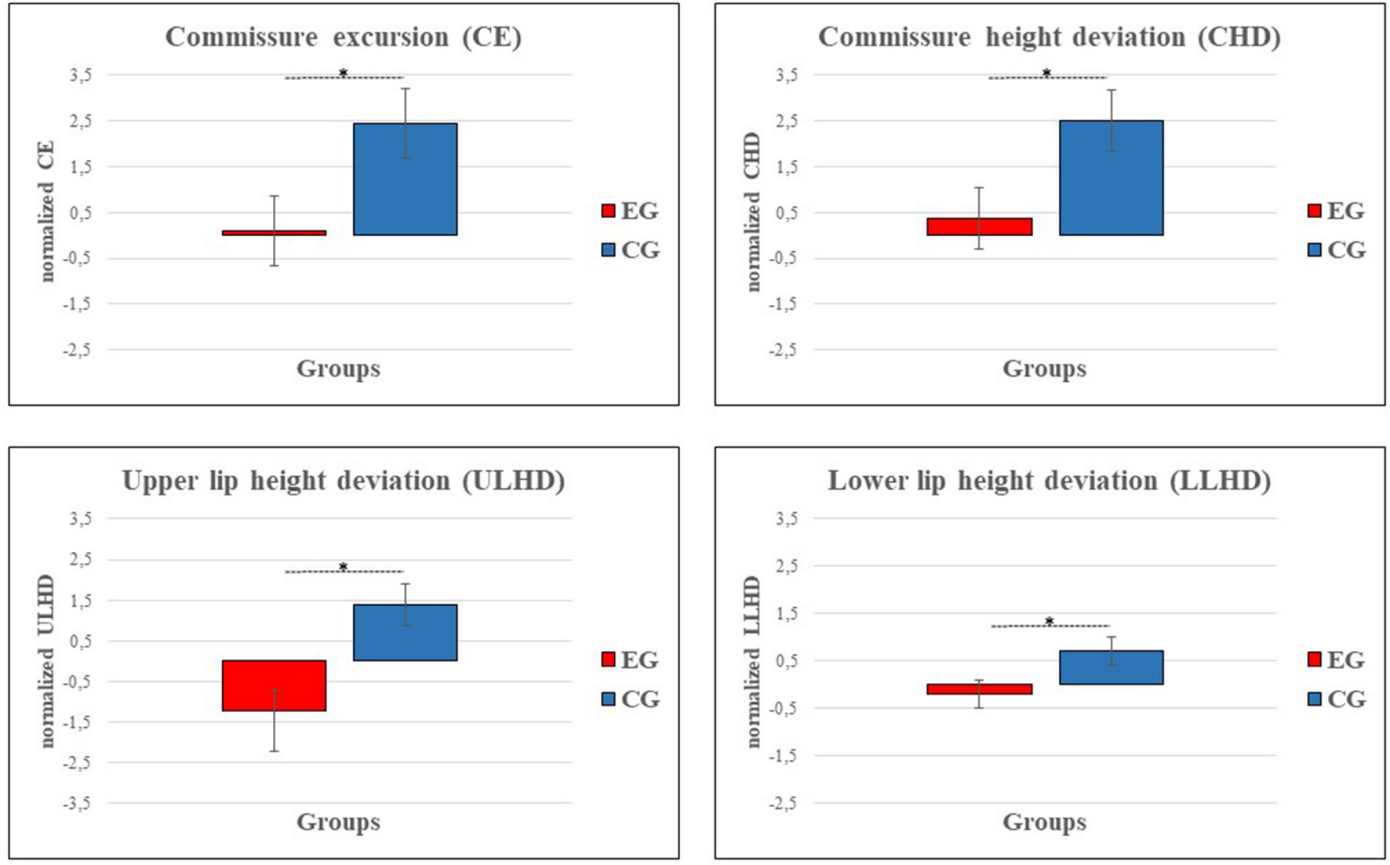
Graphs show a reduction in asymmetry between experimental (EG, red) and control group (CG, blue) in the following Emotrics measurements: commissure excursion (CE), commissure height deviation (CHD) and upper/lower lip height deviation (LLUD, CE, CHD and LLHD are represented as the difference of gentle smile values with respect to static posture. ULHD results are represented as the difference of gentle smile and maximum smile values with respect to static posture. Error bars represent SE (standard errors of the means).

Patients were divided into two groups: experimental group (EG, 4 males, mean age = 21 ± 14 years) and control group (CG, 6 males, mean age = 20 ± 9 years, see Table 1). EG rehabilitated their smile with FIT-SAT whereas CG underwent traditional treatment. Each dependent variable (CE, CHD, ULHD/LLUD and SA) was analyzed separately and statistical analysis was conducted separately for each posture (gentle and maximum smile). ANCOVAs conducted on Emotrics measurements showed a significant group effect on CE, CHE and LLHD in gentle smile only (Figure 2). Specifically, EG showed a lower values of CE (F_(1, 27)_ = 4.68, *p* = 0.039, η2 = 0.15; EG = 0.1 ± 0.7, CG = 2.4 ± 0.7), CHD (F_(1, 27)_ =5.04, *p* = 0.033, η2 = 0.18; EG = 0.37 ± 0.7, CG = 2.5 ± 0.7) and LLUD (F_(1, 27)_ = 5.24, *p* = 0.03, η2 = 0.16; EG = −0.2 ± 0.3, CG = 0.72 ± 0.3) in comparison with CG proving to obtain a better symmetry when the task requested greater control of contraction force of the transplanted muscle/s (gentle smile). A group difference was also found in ULHD regardless of the type of smile. Both movements resulted more symmetrical in the experimental group than in the control group (gentle smile: F_(1, 27)_ = 12.77, *p* = 0.001, η2 = 0.32; EG = −1.2 ± 0.5, CG = 1.4 ± 0.5; maximum smile: F_(1, 27)_ = 5.19, *p* = 0.031, η2 = 0.16; EG = −0.96 ± 0.7, CG = 1.3 ± 0.6). No differences were observed in the SA. No significant differences were observed in the FDI scores between groups (*p* > 0.05) and on average, all patients achieved similar values in physical and social function (78 ± 16 and 73 ± 19, respectively).

## Discussion

Face is the most important part of the human's body from a communicative perspective (34, 35). In particular, the ability to smile is crucial for face-to-face interactions and good smile reciprocity enhances positive social outcomes (35, 36). Facial palsy, which results in marked facial disfigurement and lack of spontaneous expressions, strongly interferes in social interactions (1, 37–39) and has a negative impact on quality of life and the overall wellbeing of patients. Thus, it is crucial that patients with facial paralysis undergoing SS can achieve a socially functional smile in terms of movement control and symmetry as soon as possible. After SS, the nerve impulse that triggers the smile comes from the masseter nerve (a branch of the trigeminal nerve) (2, 4, 6), resulting in a strong motor impulse (40). Consequently, the muscle recruitment through teeth clenching turns out to be highly effective (14) and for this reason it is commonly used in traditional post-surgery rehabilitation (14, 15). However, teeth clenching treatment presents problems related to teeth-mouth dissociation defined as the difficulty to smile independently from the original movement controlled by the donor nerve, which is teeth clenching in the case of masseteric nerve. This results in undesired teeth contraction while smiling which is difficult to avoid and, consequently, patients presents difficulties in adapting the smile to social situations. Moreover, the quality of a smile does not only depend on the extent of the excursion but also on the ability to smile symmetrically which depends on the ability to control the contraction force of the muscles (15) (smile control during excursion). For these reasons, we have proposed a new neurorehabilitation treatment (8, 15) based on action observation therapy (8, 16, 17, 41), which exploits the visuomotor coupling properties of the mirror neuron system (MNS) (19–21, 42, 43) as well as the motor synergies between the hand and the mouth present at a cortical level (24, 27, 28, 44, 45) to facilitate the recruitment of transplanted muscles.

Here we aimed at testing the efficacy of the FIT-SAT in comparison to traditional treatment in enhancing smile control in patients with facial paralysis who underwent SS.

A specific characteristic of the FIT-SAT is the use of hand contraction instead of teeth clenching. By exploiting the hand-mouth synergies present at the cortical level (24, 46), making a full fist would facilitate the recruitment of the transplanted muscle avoiding the unsightly effects related to teeth clenching (15). Specifically, some ethologically relevant human behaviors such as “hand-to-mouth goal-directed movements” (for example grasp to eat), are represented at cortical level as coordinated movements within a common control loop so that the activation of first effector (the closing of the hand) would facilitate the subsequent activation of the second effector (the opening of the mouth) (24, 27). Thus, in the FIT-SAT treatment, when patients close their hands this would facilitate oral commands toward the opening mouth (8, 15). In addition, the specific modulation exercises provided by the FIT-SAT would allow the patient to become aware of the muscle/s contraction force. Specifically, patients are asked to perform in sequence smile exercises in maximum and gentle excursion. Such exercises aim at refining the ability of patients to control smile contraction at two different levels of difficulties which require on the one hand to exert a maximum force on the transplanted muscle, and on the other hand to exert a subtler control on it. This latter exercise it is more difficult to perform because requires a modulation of motor outputs of nerve signals on the transplanted muscle.

Furthermore, the use of videos that patients firstly observe and then imitate (avoiding the use of mirror feedback) guaranteed greater control in the correct sequence of exercises and increased compliance of patients who do not like to see their own reflected image (32, 47).

Analyzing patients' photos, two main results were obtained: (1) in the maximum smile both treatments did not differ in terms of symmetry; (2) on the contrary, in the gentle smile the EG group obtained a better symmetry than the CG. These results suggested that the FIT-SAT allows patients to better modulate the smile increasing their awareness of the strength required to contract the muscles of the face. This, in turn, allows to control the extent of the lips excursion and adapt the smile to different social contexts. This is a crucial aspect to improve social interactions and one's emotions expressions through the face. Smiling indicates an intention to communicate with others and its expression varies according to the circumstances (48–50). For example, when we meet a person for the first time, a gentle smile is a precise social signal with a “welcoming” meaning. Conversely, a maximum smile can be exhibited in amusing situations or performed with an aggressive motivation (laughing at oneself or mocking). To reach an interlocutor correctly, the smile needs to not show elements of ambiguity within the context. Thus, a natural smile can present morphological characteristics that vary among different social contexts but it is fundamental that symmetry is maintained between left and right lip corners. If there is a drastic difference between the left and right lip corners (extensive asymmetry) this can not only detract from beauty and cause one's face to look unbalanced but it can also affect an interlocutor's subjective experience and emotional massage interpretation (48). A large body of studies have found that facial symmetry is an important visual cue intimately correlated with attractiveness (51, 52) having a significant impact on the social status and quality of life (53). The purpose of the modulation exercises included in the FIT-SAT is to promote the achievement of a smile with different morphological characteristics maintaining the symmetry of lips excursion.

Finally, the results of IT-FDI (32) (a self-assessment tool for measuring functional impairment and quality of life in patients with facial palsy) did not show differences between the two treatments in neither scales. The FIT-SAT reaches therefore similar criteria of patients' satisfactions in terms of quality of life compared to traditional treatments. This legitimates the use of the FIT-SAT as a post-SS rehabilitation treatment.

### Limitations of the Study

This study has some limitations. First, because of the rarity of the syndrome (54), we could only include a small number of participants, and this precludes generalization of our results. For future studies, the research question should be addressed in a larger sample. Furthermore, for reasons related to clinical practice randomization was not applied. In the future it will be necessary to include more patients and evaluate whether the efficacy of the treatment is maintained over time, for example by assessing after some years whether symmetry here obtained will remain even when the patient no longer carries out daily exercises.

## Data Availability Statement

The raw data supporting the conclusions of this article will be made available by the authors, without undue reservation.

## Ethics Statement

The studies involving human participants were reviewed and approved by the Joint Ethics Committee of the Parma Department of Medicine and Surgery and the Parma Hospital on the 12th October 2016 (Prot. 34819). Written informed consent to participate in this study was provided by the participants' legal guardian/next of kin. Written informed consent was obtained from the individual(s) for the publication of any potentially identifiable images or data included in this article.

## Author Contributions

ED, AB, and PF conceived of the presented idea and planned the experiments. AB and PF developed the theoretical framework. ED, AB, PF, MBel, CZ, CB, and RC designed and performed the experiments. AB, CZ, MBel, CB, RC, BB, AF, FZ, and MBer contributed to sample preparation and to the interpretation of the results. ED and AL performed the analysis and designed the figures. ED took the lead in writing the manuscript. ED, AB, CZ, CB, RC, AL, PS, and PF worked on the manuscript. All authors provided critical feedback and helped to shape the research analysis and manuscript.

## Funding

This research was supported by Fondazione Cariparma, Centro Diagnostico Europeo Dalla Rosa Prati e Fondazione Filippo Bassignani.

## Conflict of Interest

The authors declare that the research was conducted in the absence of any commercial or financial relationships that could be construed as a potential conflict of interest.

## Publisher's Note

All claims expressed in this article are solely those of the authors and do not necessarily represent those of their affiliated organizations, or those of the publisher, the editors and the reviewers. Any product that may be evaluated in this article, or claim that may be made by its manufacturer, is not guaranteed or endorsed by the publisher.
